# Re-positioning of known drugs for Pim-1 kinase target using molecular docking analysis

**DOI:** 10.6026/97320630015116

**Published:** 2019-02-28

**Authors:** Housna Arrouchi, Wiame Lakhlili, Azeddine Ibrahimi

**Affiliations:** 1 Biotechnology Laboratory (Medbiotech),BioInova Research center,Rabat Medical and Pharmacy School,Mohammed V University in Rabat,Rabat,Morroco

**Keywords:** drug re-profiling, Pim-1 kinase, small kinases inhibitors

## Abstract

The Concept of reusing existing drugs for new targets is gaining momentum in recent years because of cost-effectiveness as safety and toxicology data are already available. Therefore, it is of interest to re-profile known drugs against the Pim-1 kinase target using molecular docking analysis. Results show that known drugs such as nilotinib, vemurafenib, Idelalisib, and other small kinases inhibitors have high binding ability with Pim-1 kinase for consideration as potential inhibitors.

## Background

Drug re-profiling or Drug repositioning is a term for the reuse of approved substances in a new therapeutic indication [1]. This concept is popular because of its cost-effectiveness: the safety and toxicology studies are already carried out, and the results at least some parts of it-can be reused [2]. In the repositioning context, there is more rich information, like side effects, known indications, already known molecular targets and so on. There was several serendipitous repositioning in the history of drug design [3,4]. A well-known example is the case of sildenafil, which originally was developed as a cardiac medication (against angina pectoris and hypertension) and later marketed under the trade name Viagra, as an erectile dysfunction drug. The common feature of the two indications is targeted by the vasodilator property of the drug, mediated by its inhibitory effect of a phospho-di-esterase enzyme subtype (PDE5) [5].

Computational drug repositioning or repurposing is a promising and efficient tool for discovering new uses from existing drugs and holds the great potential for precision medicine in the age of big data [6,7]. Here, we are interested repositioning concept to reuse existing drugs for new targets, such a concept has been used successfully for some targets [8], we are using it for Pim-1 kinase with our own methodology. PIM kinases are constitutively active and their activity supports in vitro and in vivo tumor cell growth and survival through modification of an increasing number of common as well as isoform-specific substrates including several cell cycle regulators and apoptosis mediators. Pim-1but not Pim-2 seems also to mediate homing and migration of normal and malignant hematopoietic cells by regulating chemokine receptor surface expression [9,10].

Pim-1 belongs to a family of serine/threonine protein kinases that are highly conserved through evolution in multi-cellular organisms. Originally identified from Moloney murine leukemia virus (MuLV)-induced T-cell lymphomas in mice, Pim-1 kinase is involved in the control of cell growth, differentiation, and apoptosis. Expression of Pim-1 kinase can be stimulated by a variety of growth factors and regulated at four different levels: transcriptional, post-transcriptional, translational and posttranslational. Several signal transduction pathways may be associated with the regulation of Pim-1's expression; accumulating data support that the expression of Pim-1 protein is mediated through activation of JAK/STATs [11]. The Pim-1 oncogene is regulated by hematopoietic cytokine receptors, encodes a serine/threonine protein kinase, and cooperates with c-myc in lymphoid cell transformation [12]. Pim-1 is over expressed in human cancer diseases and has been associated, with metastasis and overall treatment response; in experimental models, inhibition of Pim-1 suppressed cell proliferation and migration, induced apoptotic cell death and synergized with other chemotherapeutic agents [13]. To our knowledge, no inhibitor clearly designed to inhibit Pim-1 is currently marketed. In particular, none of the listed molecules in the marketed drugs and clinical phase II/III sections have been developed as preclinical or clinical Pim-1 inhibitors. Most of the anti-Pim-1 developed drug candidates are in preclinical stages. However, some FDA-approved or clinical molecules, developed to inhibit other kinases, were evaluated against Pim-1 given its interest. Focusing on FDA approved Small Molecule ATPcompetitive kinase inhibitors, in this paper, we used chemical structure-based approaches and in silico strategy to investigate some property of the "chemically excited and approved" molecule with Pim-1 kinase as a new target in the goal to identify a potential Pim-1 kinase. The aim of this study is to identify candidate inhibitors for the Pim-1 kinase using docking approach and enzyme inhibition assay. We were able to identify at least four small kinases inhibitors to be inhibitors through this drug re-profiling study.

## Methodology

### Marketed drugs and PIM crystal:

The Co-crystal of the Marketed drugs ligands used in docking was obtained from the PDB database to select 3D structure of the ligands. All three-dimensional structures of the Pim-1 kinase available on the Protein Data Bank (PDB) were analyzed and classified according to the following criteria: organism, resolution, R-factor, ligand co-crystallised as ATP-competitive inhibitor; subsequently, the selection of the best crystals was made. Otherwise, structure of Cabozantinib, Regorafenib, Cobimetinib, Trametinib, Osimertinib were not available in the Protein Data Bank (PDB), their 2D structure in sdf file were extracted from the PubChem database (pubchem.ncbi.nlm.nih.gov), subsequently transformed into 3D by open babel tool v2.3.2.

### Docking methodology and docking analyses

**Figure 1:**
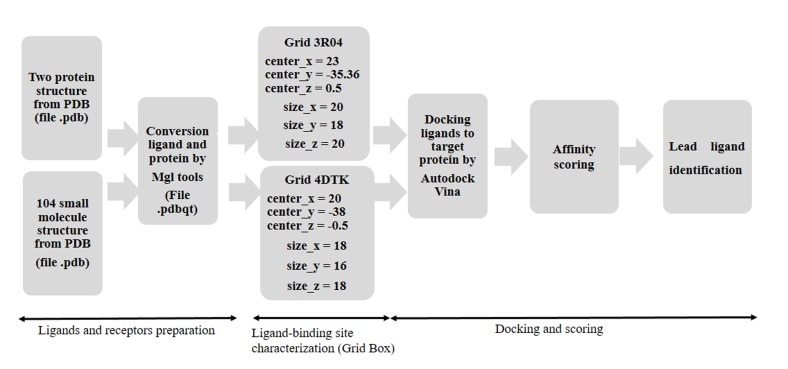
The docking strategy

MGL tools 1.5.6 with AutoGrid4 and AutoDock vina (Scripps) were used for docking studies. The Pim-1 structure was hydrogenated using the Protonate 3D in MGL tools and PyMol (DeLano Scientific) (www.pymol.org/funding.html), was used to visualize the results. The docking strategy as shown in Figure 1, involved two steps. The first one involved the preparation of elements for docking. The docking by Autodock vina requires inputs into '.pdbqt' format. The Mgltools is used to transform the ligands and receptor in.pdb format on .pdbqt format. In the same way, this program was used to determine the parameters to run the docking including the gasteiger charges, polar hydrogens, and the grid-box dimensions. The second step was the Docking itself followed by visualization by PyMol v.0.99 software. Grids box were generated around the active site of the two three-dimensional structures of the Pim-1 kinase protein using MGL tools 1.5.6. The grids box were set to have between 16 and 20Å of edge with coordinates x=23, y= -35.36, and z = 0.5 for 3R04 and x=20, y = -38, and z = -0.5 for 4DTK, both coordinates were determined using the potential substrate binding residues as centroids (in the hinge region and the activation loop). Crystallized ligands with selected Pim-1 structures were docked with all the ligands to validate the active site, these ligands are named L-pim-1 and L-pim-2 in Table 1.

## Results

**Figure 2:**
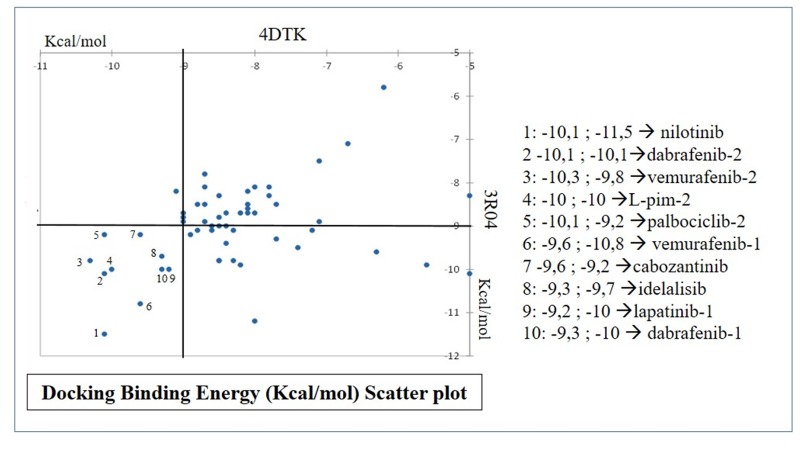
Scatter plot of FDA approved Small Molecule ATP-competitive kinase inhibitors docked into Pim-1 kinase. Nilotinib, vemurafenib, Idelalisib, lapatinib, dabrafenib, cabozantinib, palbociclib were ranked as best compounds.

The two selected crystalline structure of the Pim-1 protein (ID PDB: 4DTK and 3R04) were taken from the PDB database for molecular docking. Molecular docking was performed on the 31 marketed drugs with their different existing structures in the PDB database, which resulted in a large number of docked ligands with the two Pim-1 selected crystals. Visualization and analysis of the different interactions between ligand and receptor were made by the software Pymol v0.99. A classification was carried out for all the poses of the different docked ligands in the two crystals, this classification was done according to the best pose that presents the best energy, and the maximum of binding as it is presented on Table 1. In order to identify the ligands with a docking energy less than or equal to -9 Kcal/mol in both receptors, a ranking of the results of the two receptors was made and the graphical representation of the results as shown in Figure 2 by a scatter plot using XLSTAT (trial version, dec2018). The identified compounds are Nilotinib, vemurafenib, Idelalisib, lapatinib, dabrafenib, cabozantinib, palbociclib, as well as ligand crystallized with Pim-1 (L-pim-2).

## Discussion

**Figure 3:**
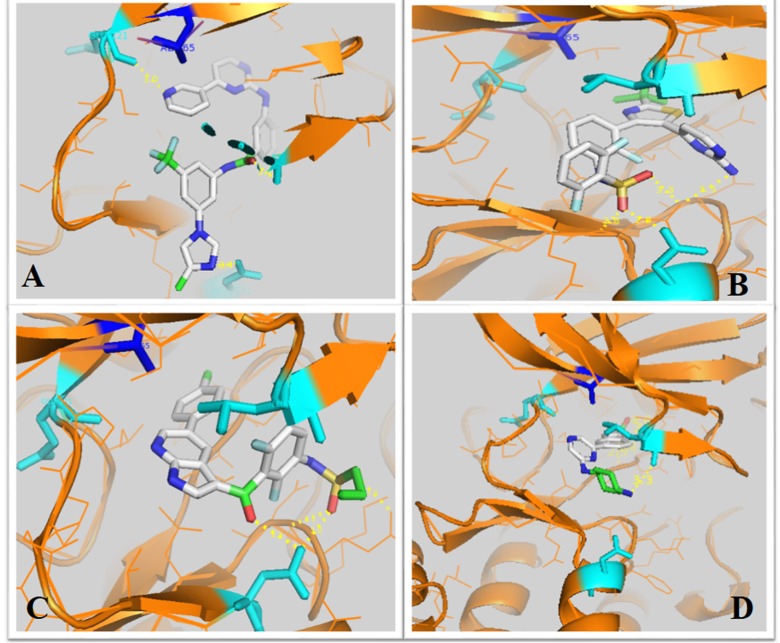
Docking mode of Nilotinib, Dabratinib and Vemurafenib to the ATP binding site of Pim-1. We selected the three inhibitors with higher hits in docking simulations and hydrogen bonds in the ATP binding site. A) Nilotinib, B) Dabrafenib, C) Vemurafenib and the experimental ligand of Pim-1 (L-pim-2)(D) Inhibitors (Nilotinib, Dabrafenib, Vemurafenib) are shown in stick representation. The hydrogen bonds between inhibitors and Pim-1 residues are indicated with yellow broken lines, Pim-1 residues (Glu121, Leu 44 and Aps131) in Cyan and Gatekeeper residue Ala65 in blue. Please see Table 1 for more details.

Drug repositioning that aims to find new uses for existing drugs is considered as an effective and alternative paradigm of drug development [14]. Computational drug repositioning provides a systematic and rational solution for identifying treatment options as compared with conventional drug repositioning approaches arising from serendipity or close clinical observation [15]. Here, we used molecular docking as in silico repurposing strategy of smallmolecule kinase inhibitors approved drugs in Pim-1 kinase as new a target. Taking advantage of the large number of Pim-1 protein structures and small-molecule kinase inhibitors available from the Protein Data Bank, a survey has been applied to discriminate between different drug-target interactions. 104 structures were docked into two best crystal of the Pim-1 kinase (4DKT, 3R04). Seven compounds, which are Nilotinib, vemurafenib, Idelalisib, lapatinib, dabrafenib, cabozantinib, palbociclib, were identified presenting the best energy and the maximum hydrogen bonds in comparison with the experimental ligand of Pim-1 (named L-pim-2) this ligand was included as a control for our docking strategy. The high docking score of all inhibitors with two three-dimensional structures of the Pim-1 can be due to Gatekeeper residue Ala65 since its small side chain can give access to the Hydrophobic Pocket II [16]. The docking mode (Figure 3) of Nilotinib, Dabratinib, and Vemurafenib with Pim-1 (PDB code 4DKT) identified interactions of the hydrogen bonds with the residues of the ATP binding site. The docking results of the other inhibitors with Pim-1 have been grouped in Table 1.

## Conclusion

Drug repositioning has economic and public health benefits for drug makers, regulatory agencies and patients. A rational way to search for repositioning opportunities is an important step in optimizing the drug discovery pipeline. Enormous amount of data generated by various techniques, in different formats and diverse domains are available. Optimized tools to retrieve, organize and mine these resources effectively for drug repositioning is required. We show the use of known drug re-positioning using molecular docking analysis against Pim-1 kinase.

## Author’s contributions:

All authors designed research. H.A and W.L experimentation and wrote the manuscript and analyzed data. A.I is the principal investigator. All authors read and approved the final manuscript.

## Conflict of Interest

We report no conflicts of interest in this work.

## Figures and Tables

**Table 1:** The docking results the Small Molecule ATP-competitive kinase inhibitors with Pim-1

Ligands	Pose	Number of interacting bonds	Residues in 4DKT (distance in Å)	Residues in 3R04 (distance in Å)	Docking binding energy (Kcal/mol)	
4DTK	3R04
Nilotinib	3	3	L44 (3,15)	D131 (3,07)		
			D131 (3,43)		-10, 1	-11, 5
			E121 (2,95)			
Dabrafenib_2	2	4	D128 (3,46+3,26)	L44 (3,41)		
			D131 (2,85)	E121 (3,09)	-10, 1	-10, 1
			D128 (2,81)			
Vemurafenib_2	1	6	E171 (3,48+3,10+3,19)	E171 (3,57+3,41)		
			D128 (3,08)	D128 (3,38+3,10)	-10, 3	-9, 8
			K67 (2,87)	E171 (2,88)		
			D186 (3,12)			
L-pim-2	1	6	L44 (3,31)	D131 (3,47)		
			K67 (3,01+3,28)	K67 (3,42+3,55)		
			E89 (2,77)	E89 (3,00)	-10	-10
			D186 (2,95)	D186 (2,91)		
			F187 (3,36)	F187 (3,25)		
palbociclib_2	1	2	L44 (3,35+2,94)	S54 (3,45)		
				R122 (3,08)	-10, 1	-9, 2
				D128 (3,28)		
Vemurafenib_1	1	4	D128 (2,98+3,28)	D128 ((3,42)		
			E171 (3,12+3,03)	E171 (3,26+3,15)	-9, 6	-10, 8
Cabozantinib	2	6	D131 (2,1+3,1)	D131 (3,36)		
			D128 (2,8)	D128 (3,38)		
			D186 (3,1)	K67 (3,53)	-9, 6	-9, 2
			K67 (3,2+3,4)	D186 (2,81)		
				V126 (3,23)		
Idelalisib	1	5	E171 (3,20+3,43+3,12)	E171 (3,43)	-9, 3	-9, 7
			D128 (3,50+3,17)	D128 (3,23)		
Lapatinib_1	4	1	D131 (3,35)	E171 (3,25)	-9, 2	-10
				N172 (3,31+3,43)		
Dabrafenib_1	1	3	D131 (2,85)	E171 (3,37)	-9, 3	-10
			D128 (2,80+3,07)	D128 (3,33)
